# Resuscitation, survival and morbidity of extremely preterm infants in California 2011–2019

**DOI:** 10.1038/s41372-023-01774-6

**Published:** 2023-09-09

**Authors:** Brennan V. Higgins, Rebecca J. Baer, Martina A. Steurer, Kayla L. Karvonen, Scott P. Oltman, Laura L. Jelliffe-Pawlowski, Elizabeth E. Rogers

**Affiliations:** 1 Division of Neonatology, Department of Pediatrics, University of California, San Francisco, San Francisco, CA USA; 2 California Preterm Birth Initiative, University of California, San Francisco, San Francisco, CA USA; 3 Department of Pediatrics, University of California San Diego, San Diego, CA USA; 4 Department of Epidemiology and Biostatistics, University of California, San Francisco, CA USA

**Keywords:** Paediatrics, Risk factors

## Abstract

**Objective:**

To describe changes over time in resuscitation, survival, and morbidity of extremely preterm infants in California.

**Study design:**

This population-based, retrospective cohort study includes infants born ≤28 weeks. Linked birth certificates and hospital discharge records were used to evaluate active resuscitation, survival, and morbidity across two epochs (2011–2014, 2015–2019).

**Results:**

Of liveborn infants, 0.6% were born ≤28 weeks. Active resuscitation increased from 16.9% of 22-week infants to 98.1% of 25-week infants and increased over time in 22-, 23-, and 25-week infants (*p*-value ≤ 0.01). Among resuscitated infants, survival to discharge increased from 33.2% at 22 weeks to 96.1% at 28 weeks. Survival without major morbidity improved over time for 28-week infants (*p*-value < 0.01).

**Conclusion:**

Among infants ≤28 weeks, resuscitation and survival increased with gestational age and morbidity decreased. Over time, active resuscitation of periviable infants and morbidity-free survival of 28-week infants increased. These trends may inform counseling around extremely preterm birth.

## Introduction

Preterm birth, defined as birth before 37 weeks completed gestation, is the second leading cause of infant death in the United States [1]. In 2019, the preterm birth rate in the United States was 10.2 per 1000 live births, an increase from 10.0 in 2018 [1, 2]. Of all births, 0.66% occur before 28 weeks gestation [2], the period defined as “extremely preterm.” Extremely preterm infants are at greatest risk of mortality and morbidity with an inverse relationship between gestational age and risk [3, 4]. With time, there has been a national trend towards improved survival and reduced morbidity of this vulnerable population [4–7] with attention to modifiable clinical risk factors such as antenatal corticosteroids [8]. The impact of non-clinical risk factors including race/ethnicity, socioeconomic status (SES), and maternal education, known to impact preterm birth and outcomes in the United States [9–15], are less clear in this population [16–18].

Of particular focus are infants considered to be born at the limit of viability. Active resuscitation is offered starting between 22–24 weeks gestation, with considerable international, national and institutional variation [17, 19, 20]. In 2014, the Eunice Kennedy Shriver National Institute of Child Health and Human Development, Society for Maternal-Fetal Medicine (SMFM), American Academy of Pediatrics (AAP), and American College of Obstetricians and Gynecologists (ACOG) held a joint workshop to summarize best practices for management during the periviable period (<26 weeks gestational age) [21]. The workshop described more active management at a younger gestation than had been recommended previously and advocated that at 22–23 weeks management decisions should be made based on individual clinical circumstances and family preferences [21]. Research priorities from this workshop included updated population-based cohort studies on outcomes to guide clinicians and families [21].

The primary aim of this study, therefore, is to provide updated rates of resuscitation, survival, and major morbidity by gestational age for preterm infants born between 22 and 28 weeks completed gestation in California between 2011 and 2019. Secondary aims include assessing demographic, antenatal, perinatal, and infant characteristics of this group and changes in resuscitation patterns, survival, and major morbidity over time.

## Methods

**Fig. 1:**
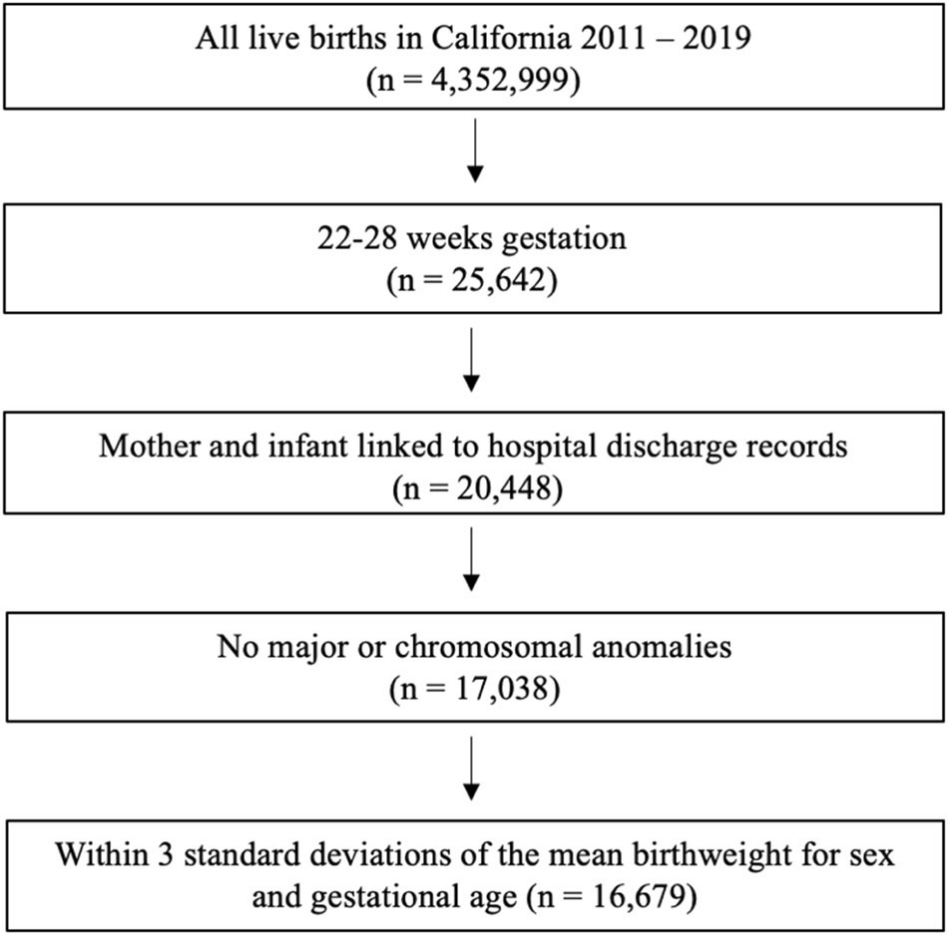
Sample selection.

The study sample was drawn from all live born infants in California between 2011 and 2019. The sample was restricted to infants born between 22 and 28 weeks completed gestation (as determined by best obstetric estimate reported on birth certificate records), infants whose birth records could be linked to maternal records, and infants without major or chromosomal anomalies. Anomalies were considered “major” if determined by expert review to cause mortality or major morbidity that would likely be identified at birth or lead to hospitalization during the first year of life [22]. Additionally, to eliminate implausible birthweight and gestational age combinations, infants with a birthweight for sex outside of three standard deviations of the mean [23] were removed from the sample (Fig. 1).

Birth certificates, which included linked infant deaths, maintained by California Vital Statistics, were linked [24] to hospital discharge records maintained by the California Department of Health Care Access and Information. If discharge status indicated transfer, the next admission was assumed to be the transfer record and included. Demographic, antenatal, perinatal and infant characteristics were drawn from both birth certificates and discharge records. Hospital discharge records additionally provided diagnosis and procedure codes based on the International Classification of Diseases (ICD) United States Clinical Modification, ninth and tenth revision [25, 26] (Supplementary Appendix 1).

Race was self-identified by parents. County of birth was used to classify the mother’s county of residence as urban or rural according to Federal Information Processing Standard Publication codes. Hospital of birth was used to stratify birthplace into Northern California (zip code prefixes 90–93) and Southern California (prefixes 94–96) and to classify neonatal intensive care unit (NICU) level. NICU level was defined using the California Children’s Services Department certification as intermediate (able to provide short-term ventilatory assistance), community (able to provide long-term ventilatory assistance), or regional (full range of services including neonatal surgery) [27, 28].

Active resuscitation was defined as a documented ICD code for cardiopulmonary resuscitation, intubation, invasive mechanical ventilation, or non-invasive mechanical ventilation (Supplementary Appendix 1). Other recent studies have included additional procedures or therapies in the definition of active care such as administration of surfactant, parenteral nutrition, epinephrine, vasopressors, antibiotics, and/or volume resuscitation [6, 8, 18, 29]. These additional measures were not included in our definition as all extremely preterm infants receiving active care should receive respiratory support and thus be captured with the more simple definition. Additionally, infants who did not have one of the above ICD codes in their record but who survived for more than 24 h were included in our active resuscitation group. Relatively few infants (7% of sample) were included based on 24-h survival alone. Nevertheless, we feel that in the setting of extreme prematurity, where survival is dependent on active care including respiratory support, this is a valid assumption and is important for capturing infants who may otherwise have been missed due to ICD coding errors. Additionally, it allows this cohort study to be comparable in methodology to a previously published statewide cohort (2007–2011) [30].

Death was obtained from birth certificates linked to infant death certificates or when the discharge status reflected death. Conversely, survival was assumed when there was no linked infant death certificate or indication of discharge status equaling death. There were 56 infants who died before 28 days and had an indication on their birth hospital record that they were discharged home. Given the inability to be discharged home alive at <28 days old when born ≤28 weeks, it was assumed that these infants were incorrectly coded as discharged rather than deceased. They were considered to have died before discharged for the purpose of this analysis. Major morbidity was defined as intraventricular hemorrhage (IVH) grade III or IV, periventricular leukomalacia (PVL), necrotizing enterocolitis (NEC), bronchopulmonary dysplasia (BPD), retinopathy of prematurity (ROP) requiring intervention, or sepsis and was assessed using ICD codes (Supplementary Appendix 1).

The study period was divided into two epochs (2011–2014 and 2015–2019) to assess change in practice and outcomes over time. The second epoch begins after the Eunice Kennedy Shriver National Institute of Child Health and Human Development, SMFM, AAP, and ACOG joint workshop [21]. The second epoch also reflects the most recent five years prior to the COVID-19 pandemic.

The two-sided Cochrane-Armitage test for trend was used to evaluate demographic, antenatal, perinatal, and infant characteristics across the included gestational age spectrum of 22–28 weeks. Chi-square statistics were used to identify statistical differences in active resuscitation, survival amongst resuscitated infants, and major morbidity amongst infants surviving to hospital discharge between the two epochs by gestational age. All analyses were performed using Statistical Analysis Software, version 9.4 (Cary, NC).

Methods and protocols for the study were approved by the Committee for the Protection of Human Subjects within the Health and Human Services Agency of the State of California. This work was supported by the California Preterm Birth Initiative within the University of California, San Francisco.

## Results

### Descriptive characteristics

Of the 4.35 million live deliveries in California from 2011–2019, 0.6% were born between 22- and 28-weeks gestation (*n* = 25,642) and, of those, 65% met the study inclusion criteria (sample size 16,679 infants) (Fig. 1). Approximately 30% of deliveries occurred in Northern California and 70% in Southern California. Additionally, approximately 60% of infants were born to mothers living in the most urban areas of California and 5% to individuals living in the most rural areas. The rate of cesarean delivery increased with gestational age from 13.3% at 22 weeks to 75.1% at 28 weeks (*p*-value < 0.01) (Table 1).

**Table 1:** Demographic, antenatal, perinatal, and infant characteristics.

Characteristic	Gestational age in weeks							
	22	23	24	25	26	27	28	*p*-value^a^
Sample	1158	1523	2132	2244	2734	3048	3840	
Birth weight, g								
Mean	486.8	570.3	666.4	761.6	876.0	1001.0	1128.7	
SD	82.7	96.6	121.1	141.8	166.6	193.8	219.0	
Male	625 (54.0)	790 (51.9)	1097 (51.5)	1133 (50.5)	1430 (52.3)	1499 (49.2)	1955 (50.9)	0.05
Singleton birth	913 (78.8)	1193 (78.3)	1643 (77.1)	1786 (79.6)	2140 (78.3)	2326 (76.3)	2836 (73.9)	<0.001
Cesarean delivery	154 (13.3)	669 (43.9)	1462 (68.6)	1623 (72.3)	1997 (73.0)	2271 (74.5)	2882 (75.1)	<0.001
No prenatal visits	54 (4.7)	66 (4.3)	72 (3.4)	76 (3.4)	64 (2.3)	80 (2.6)	108 (2.8)	<0.001
Diabetes								
Preexisting	27 (2.3)	48 (3.2)	60 (2.8)	79 (3.5)	85 (3.1)	109 (3.6)	142 (3.7)	0.02
Gestational	91 (7.9)	120 (7.9)	211 (9.9)	221 (9.9)	349 (12.8)	405 (13.3)	589 (15.3)	<0.001
Obesity	359 (31.0)	471 (30.9)	645 (30.3)	691 (30.8)	771 (28.2)	895 (29.4)	1087 (28.3)	0.01
Hypertension								
Preexisting	42 (3.6)	66 (4.3)	74 (3.5)	104 (4.6)	80 (2.9)	122 (4.0)	129 (3.4)	0.24
Gestational	12 (1.0)	28 (1.8)	54 (2.5)	48 (2.1)	64 (2.3)	98 (3.2)	120 (3.1)	<0.001
Preeclampsia	35 (3.0)	72 (4.7)	197 (9.2)	299 (13.3)	478 (17.5)	637 (20.9)	903 (23.5)	<0.001
Chorioamnionitis	210 (18.1)	267 (17.5)	360 (16.9)	314 (14.0)	356 (13.0)	336 (11.0)	327 (8.5)	<0.001
Maternal Age								
<18 y	26 (2.3)	24 (1.6)	51 (2.4)	56 (2.5)	48 (1.8)	41 (1.4)	64 (1.7)	0.03
18–34 y	887 (76.6)	1149 (75.4)	1578 (74.0)	1616 (72.0)	1967 (72.0)	2160 (70.9)	2731 (71.1)	<0.001
>34 y	245 (21.2)	347 (22.8)	503 (23.6)	572 (25.5)	718 (26.3)	847 (27.8)	1045 (27.2)	<0.001
Maternal Education								
<12 y	185 (16.0)	243 (16.0)	350 (16.4)	402 (17.9)	475 (17.4)	517 (17.0)	630 (16.4)	0.64
12 y	285 (24.6)	392 (25.7)	570 (26.7)	590 (26.3)	693 (25.4)	785 (25.8)	996 (25.9)	0.86
>12 y	525 (45.3)	721 (47.3)	1057 (49.6)	1113 (49.6)	1396 (51.1)	1572 (51.6)	1998 (52.0)	<0.001
Race/ethnicity								
White, non-Hispanic	194 (16.8)	279 (18.3)	393 (18.4)	424 (18.9)	489 (17.9)	612 (20.1)	814 (21.2)	0.001
Hispanic	540 (46.6)	738 (48.5)	1075 (50.4)	1090 (48.6)	1358 (49.7)	1431 (47.0)	1821 (47.4)	0.27
Black	162 (14.0)	195 (12.8)	262 (12.3)	294 (13.1)	357 (13.1)	352 (11.6)	456 (11.9)	0.04
Asian	130 (11.2)	150 (9.9)	242 (11.4)	259 (11.5)	339 (12.4)	435 (14.3)	498 (13.0)	<0.001
Other	129 (11.1)	161 (10.6)	158 (7.4)	176 (7.8)	188 (6.9)	214 (7.0)	249 (6.5)	<0.001
Born outside the US	375 (32.4)	518 (34.0)	709 (33.3)	733 (32.7)	949 (34.7)	1111 (36.5)	1327 (34.6)	0.02
WIC participation	454 (39.2)	687 (45.1)	1008 (47.3)	1082 (48.2)	1334 (48.8)	1464 (48.0)	1797 (46.8)	0.001
Insurance Status								
Private	504 (43.5)	658 (43.2)	959 (45.0)	1011 (45.1)	1292 (47.3)	1410 (46.3)	1839 (47.9)	<0.001
Medi-Cal	581 (40.2)	750 (49.2)	1057 (49.6)	1136 (50.6)	1306 (47.8)	1472 (48.3)	1772 (46.2)	0.001
Other	73 (6.3)	115 (7.6)	116 (5.4)	97 (4.3)	136 (5.0)	166 (5.5)	229 (6.0)	0.26
Birth Hospital NICU								
No NICU	295 (25.5)	304 (20.0)	390 (18.3)	417 (18.6)	435 (15.9)	510 (16.7)	640 (16.7)	<0.001
Intermediate	42 (3.6)	65 (4.3)	97 (4.6)	71 (3.2)	93 (3.4)	110 (3.6)	133 (3.5)	0.13
Community	629 (54.3)	902 (59.2)	1268 (59.5)	1307 (58.2)	1647 (60.2)	1808 (59.3)	2305 (60.0)	0.01
Regional	192 (16.6)	252 (16.7)	377 (17.7)	449 (20.0)	559 (20.5)	620 (20.3)	762 (19.8)	<0.001
Birth Place								
Northern CA	329 (28.4)	425 (27.9)	584 (27.4)	671 (29.9)	811 (29.7)	938 (30.8)	1197 (31.2)	0.001
Southern CA	882 (71.0)	1092 (71.7)	1539 (72.2)	1562 (69.6)	1914 (70.0)	2099 (68.9)	2627 (68.4)	0.001
Residential county FIPS code								
1 (most urban)	648 (56.0)	893 (58.6)	1286 (60.3)	1305 (58.2)	1685 (61.6)	1874 (61.5)	2350 (61.2)	<0.001
2	166 (14.3)	190 (12.5)	245 (11.5)	310 (13.8)	338 (12.4)	405 (13.3)	519 (13.5)	0.48
3	271 (23.4)	346 (22.7)	453 (21.3)	486 (21.7)	545 (19.9)	603 (19.8)	766 (20.0)	0.001
4,5,6 (most rural)	61 (5.3)	81 (5.3)	133 (6.2)	125 (5.6)	142 (5.2)	149 (4.9)	180 (4.7)	0.05

*US* United States, *WIC* Special Supplemental Nutrition Program for Women, Infants, and Children, *NICU* Neonatal Intensive Care Unit, *CA* California, *FIPS* Federal Information Processing Standard Publication.

^a^Cochrane-Armitage test for trend (2-sided).

Regarding maternal characteristics, approximately one third of births were to mothers born outside the United States and just under half of the cohort participated in WIC (47%) or had Medi-Cal insurance (48%). With increasing gestational age, an increasing percentage of infants were born to mothers over 34 years of age (*p*-value < 0.01), with >12 years of education (*p*-value < 0.01), and identifying as White (*p*-value < 0.01). Additionally, with increasing gestational age, there were increased rates of antenatal maternal complications including gestational diabetes (*p*-value < 0.01), gestational hypertension (*p*-value < 0.01), and preeclampsia (*p*-value < 0.01). Please see Table 1 for full demographic details.

### Resuscitation

Active resuscitation was performed in 16.9% of 22-week infants, 67.8% of 23-week infants, 93.1% of 24-week infants, and 98.1% of 25-week infants. Over 99% of infants were resuscitated thereafter. Between the two study epochs, a significant increase in active resuscitation was observed in 22-week infants (*p-*value < 0.01), 23-week infants (*p-*value < 0.01), and 25-week infants (*p-*value = 0.01) (Tables 2 and 3).

**Table 2:** Resuscitation and survival for infants born between 22- and 25-weeks gestation.

	Gestational age in weeks															
	22				23				24				25			
	2011–2019	2011–2014	2015–2019	*p*-value^a^	2011–2019	2011–2014	2015–2019	*p*-value^a^	2011–2019	2011–2014	2015–2019	*p*-value^a^	2011–2019	2011–2014	2015–2019	*p*-value^a^
Sample	1158	545	613		1523	742	781		2132	1024	1108		2244	1092	1152	
Resuscitation attempted	196 (16.9)	72 (13.2)	124 (20.2)	0.002	1033 (67.8)	477 (64.3)	556 (71.2)	0.004	1984 (93.1)	949 (92.7)	1035 (93.4)	0.50	2201 (98.1)	1062 (97.3)	1139 (98.9)	0.01
Among those resuscitated																
Survival to 1 day	145 (74.0)	54 (75.0)	91 (73.4)	0.80	876 (84.8)	401 (84.1)	475 (85.4)	0.54	1844 (93.0)	887 (93.6)	957 (92.5)	0.34	2133 (96.9)	1032 (97.2)	1101 (96.7)	0.49
Survival to 28 days	70 (35.7)	31 (43.1)	39 (31.5)	0.10	516 (50.0)	240 (50.3)	276 (49.6)	0.83	1402 (70.7)	664 (70.0)	738 (71.3)	0.55	1858 (84.4)	911 (85.8)	947 (83.1)	0.09
Survival to hospital discharge	65 (33.2)	29 (40.3)	36 (29.0)	0.11	472 (45.6)	222 (46.5)	250 (45.0)	0.61	1,330 (67.1)	628 (66.2)	702 (67.8)	0.45	1797 (81.6)	876 (82.5)	921 (80.9)	0.32
Survival to hospital discharge without major morbidity	^b^	^b^	^b^	0.11	51 (4.9)	30 (6.3)	21 (3.8)	0.06	241 (12.2)	124 (13.1)	117 (11.3)	0.23	419 (19.0)	214 (20.2)	205 (18.0)	0.20

^a^Chi square test comparing epoch 1 (2011–2014) and epoch 2 (2015–2019).

^b^Not displayed when *n* < 5.

**Table 3:** Resuscitation and survival for infants born between 26- and 28-weeks gestation.

	Gestational age in weeks											
	26				27				28			
	2011–2019	2011–2014	2015–2019	*p*-value^a^	2011–2019	2011–2014	2015–2019	*p*-value^a^	2011–2019	2011–2014	2015–2019	*p*-value^a^
Sample	2734	1289	1445		3048	1384	1664		3840	1733	2107	
Resuscitation attempted	2711 (99.2)	1277 (99.1)	1434 (99.2)	0.63	3029 (99.4)	1378 (99.6)	1651 (99.2)	0.22	3827 (99.7)	1728 (99.7)	2099 (99.6)	0.63
Among those resuscitated												
Survival to 1 day	2668 (98.4)	1258 (98.5)	1410 (98.4)	0.70	3001 (99.1)	1362 (98.8)	1639 (99.3)	0.21	3790 (99.0)	1716 (99.3)	2074 (98.8)	0.12
Survival to 28 days	2490 (91.9)	1181 (92.5)	1309 (91.3)	0.25	2886 (95.3)	1314 (95.4)	1572 (95.2)	0.86	3696 (96.6)	1671 (96.7)	2025 (96.5)	0.70
Survival to hospital discharge	2429 (89.6)	1152 (90.2)	1277 (89.1)	0.32	2850 (94.1)	1299 (94.3)	1551 (93.9)	0.71	3,676 (96.1)	1657 (95.9)	2019 (96.2)	0.64
Survival to hospital discharge without major morbidity	852 (31.4)	389 (30.5)	463 (32.3)	0.31	1335 (44.1)	583 (42.3)	752 (45.6)	0.07	2129 (55.6)	911 (52.7)	1218 (58.0)	0.001

^a^Chi square test comparing epoch 1 (2011–2014) and epoch 2 (2015–2019).

### Survival

Among those infants resuscitated at 22 weeks, 74.0% survived to 1 day, 35.7% to 28 days, and 33.2% to hospital discharge. At 23 weeks, 84.8% of infants survived to 1 day, 50.0% to 28 days, and 45.6% to hospital discharge. At 24 weeks, 93.0% of infants survived to 1 day, 70.7% to 28 days, and 67.1% to hospital discharge. At 25 weeks, 96.9% of infants survived to 1 day, 84.4% to 28 days, and 81.6% to hospital discharge. Between 26 and 28 weeks over 98% of infants survived to 1 day, 92% to 28 days, and 90% to hospital discharge. A statistically significant improvement in survival to hospital discharge without major morbidity was seen in 28-week infants between the two epochs (*p*-value < 0.01). Changes in survival over time were not observed in 22–27 week infants (Tables 2 and 3).

### Morbidity

Of 22-week infants who survived to hospital discharge, 93.9% survived with a major morbidity. At 23 weeks, 89.2% of infants surviving to hospital discharge did so with a major morbidity. From 24 to 28 weeks, the percentage of infants who suffered from major morbidity nearly halved (81.9% at 24 weeks to 42.1% at 28 weeks). A statistically significant decrease in major morbidity among survivors to hospital discharge was seen at 28 weeks between the two study epochs (*p*-value < 0.01) (Tables 4 and 5).

**Table 4:** Major morbidity in infants born between 22- and 25-weeks gestation who survived to hospital discharge.

	Gestational age in weeks															
	22				23				24				25			
	2011–2019	2011–2014	2015–2019	*p*-value^a^	2011–2019	2011–2014	2015–2019	*p*-value^a^	2011–2019	2011–2014	2015–2019	*p*-value^a^	2011–2019	2011–2014	2015–2019	*p*-value^a^
Sample	65	29	36		472	222	250		1300	628	702		1797	876	921	
IVH, grade III or IV	8 (27.6)	10 (27.8)	0.9863	0.88	67 (14.2)	32 (14.4)	35 (14.0)	0.90	151 (11.4)	77 (12.3)	74 (10.5)	0.32	156 (8.7)	78 (8.9)	78 (8.5)	0.74
PVL	^b^	^b^	^b^	0.69	22 (4.7)	7 (3.2)	15 (6.0)	0.14	53 (4.0)	28 (4.5)	25 (3.6)	0.40	60 (3.3)	24 (2.7)	36 (3.9)	0.17
NEC	6 (9.2)	^b^	^b^	0.56	61 (12.9)	22 (9.9)	39 (15.6)	0.07	158 (11.9)	72 (11.5)	86 (12.3)	0.66	170 (9.5)	84 (9.6)	86 (9.3)	0.86
BPD	38 (58.5)	5 (51.7)	23 (63.9)	0.32	288 (61.0)	120 (54.1)	168 (67.2)	0.004	732 (55.0)	324 (51.6)	408 (58.1)	0.02	811 (45.1)	369 (42.1)	442 (48.0)	0.01
ROP requiring intervention	23 (35.4)	13 (44.8)	10 (27.8)	0.15	174 (36.9)	82 (36.9)	92 (36.8)	0.98	348 (26.2)	163 (26.0)	185 (26.4)	0.87	311 (17.3)	156 (17.8)	155 916.8)	0.58
Sepsis	53 (81.5)	24 (82.8)	29 (80.6)	0.82	297 (62.9)	137 (61.7)	160 (64.0)	0.61	708 (53.2)	341 (54.3)	367 (52.3)	0.46	876 (48.8)	452 (51.6)	424 (46.0)	0.02
>1 Morbidity	45 (69.2)	21 (72.4)	24 (66.7)	0.62	300 (63.6)	131 (59.0)	169 (67.6)	0.05	675 (50.8)	314 (50.0)	361 (51.4)	0.60	697 (38.8)	343 (39.2)	354 (38.4)	0.75
Any Major Morbidity	61 (93.9)	26 (89.7)	35 (97.2)	0.21	421 (89.2)	192 (86.5)	229 (91.6)	0.07	1089 (81.9)	504 (80.3)	585 (83.3)	0.15	1378 (76.7)	662 (75.6)	716 (77.7)	0.28

*IVH* Intraventricular Hemorrhage, *PVL* Periventricular Leukomalacia, *NEC* Necrotizing Enterocolitis, *BPD* Bronchopulmonary Dysplasia, *ROP* Retinopathy of Prematurity.

^a^Chi square test comparing epoch 1 (2011–2014) and epoch 2 (2015–2019).

^b^Not displayed when *n* < 5.

**Table 5:** Major morbidity in infants born between 26- and 28-weeks gestation who survived to hospital discharge.

	Gestational age in weeks											
	26				27				28			
	2011–2019	2011–2014	2015–2019	*p*-value^a^	2011–2019	2011–2014	2015–2019	*p*-value^a^	2011–2019	2011–2014	2015–2019	*p*-value^a^
Sample	2429	1152	1277		2850	1299	1551		3676	1657	2019	
IVH, grade III or IV	122 (5.0)	51 (4.4)	71 (5.6)	0.20	126 (4.4)	63 (4.9)	63 (4.1)	0.31	69 (1.9)	29 (1.8)	40 (2.0)	0.61
PVL	40 (1.7)	11 (1.0)	29 (2.3)	0.01	47 (1.7)	18 (1.4)	29 (1.9)	0.31	57 (1.6)	25 (1.50	32 (1.6)	0.85
NEC	145 (6.0)	71 (6.2)	74 (5.8)	0.70	160 (5.6)	78 (6.0)	82 (5.3)	0.41	148 (4.0)	67 (4.0)	81 (4.0)	0.96
BPD	895 (36.9)	404 (35.1)	491 (38.5)	0.08	711 (25.0)	301 (23.2)	410 (26.4)	0.05	588 (16.0)	247 (14.9)	341 (16.9)	0.10
ROP requiring intervention	213 (8.8)	113 (9.8)	100 (7.8)	0.09	94 (3.3)	46 (3.5)	48 (3.1)	0.51	78 (2.1)	34 (2.1)	44 (2.2)	0.79
Sepsis	952 (39.2)	504 (43.8)	448 (35.1)	<0.001	966 (33.9)	487 (37.5)	479 (30.9)	<0.001	1041 (28.3)	564 (34.0)	477 (23.6)	<0.001
>1 Morbidity	614 (25.3)	303 (26.3)	311 (24.4)	0.27	477 (16.7)	221 (17.0)	256 (16.5)	0.72	364 (9.9)	182 (11.0)	182 (9.0)	0.05
Any Major Morbidity	1577 (64.9)	763 (66.2)	814 (63.7)	0.20	1515 (53.2)	716 (55.1)	799 (51.5)	0.05	1457 (42.1)	746 (45.0)	801 (39.7)	0.001

^a^Chi square test comparing epoch 1 (2011–2014) and epoch 2 (2015–2019).

*IVH* Intraventricular Hemorrhage, *PVL* Periventricular Leukomalacia, *NEC* Necrotizing Enterocolitis, *BPD* Bronchopulmonary Dysplasia, *ROP* Retinopathy of Prematurity.

Sepsis was the most common major morbidity at all gestational ages except 24 weeks when BPD affected slightly more infants. A significant decrease in the percentage of infants born between 25 and 28 weeks affected by sepsis was observed between the two study epochs (*p*-value ≤ 0.02). The rate of BPD was highest at 23 weeks (61.0%) and decreased to 16.0% at 28 weeks. A significant increase in the rate of BPD was observed in 23- to 25-week infants between the two epochs (*p*-value ≤ 0.02) (Tables 4 and 5). Rates of IVH, PVL, NEC, and ROP are presented in Tables 4 and 5.

## Discussion

This population-based study of infants born in California between 2011 and 2019 provides a representative description of how care of extremely preterm infants is evolving in the United States. In California, 0.6% of live births occurred between 22 and 28 weeks, comparable to national data [2]. The majority of extremely preterm deliveries in the state occurred in urban areas (60%), in Southern California (70%), and in hospitals with community level NICUs (59%). Percentages are comparable in term deliveries (62%, 68%, 49% respectively) and all liveborn deliveries (62%, 68%, 50% respectively) in the state. Regarding antenatal and perinatal risk factors, with increasing gestational age, there were increased rates of maternal diabetes, gestational hypertension, preeclampsia, and C-section delivery. Compared to a previous statewide population-based study (2007–2011) with similar methods [30], rates of maternal diabetes have increased substantially likely driven by gestational diabetes and consistent with national trends [31]. Rates of C-section have increased across gestational ages 23–28 weeks. Observational data suggests delivery via C-section reduces the risk of mortality and IVH for extremely preterm infants [32, 33] but the associated maternal morbidity should not be underestimated [34].

With increasing gestational age at delivery, an increasing percentage of the study sample was born to mothers over 34 years of age, with higher education levels, and self-identifying as White. It is well known that there are significant inequities in preterm birth rates in the United States [13, 14]. Black women are 50% more likely to have a preterm delivery compared to all other women [15]. Additional factors such as SES and maternal level of education play a complex, non-uniform role in the risk of preterm birth [16, 17] but do not explain underlying racial disparities [14] or account for the health impacts of systemic racism [35–37]. Although the role of these factors is less clear in extremely preterm delivery [16–18], differential risk of extremely preterm delivery cannot be excluded as an explanation for the patterns observed in this study. Alternatively, the data may reflect variation in active treatment decisions. Previously published literature supports racial/ethnic as well as SES differences in neonatal intervention in the periviable period. Studies have demonstrated infants born to non-White mothers of lower SES receiving more intervention at the extreme end of periviability [18, 29, 38] as well as the opposite [39]. This is an important area for further study and an area of active inquiry of our group.

The percentage of liveborn infants in this study receiving active resuscitation increased with increasing gestational age with the most substantial change in practice patterns occurring between 22 weeks (17% resuscitated) and 24 weeks (93% resuscitated). Additionally, there was a significant increase in active resuscitation of 22-, 23- and 25-week infants in the latter half of the study period compared to the earlier epoch. For 22-week infants, this increase represented an increase back up to the statewide rate observed from 2007–2011 [30] after a relative decline in resuscitation rates from 2011–2014. Resuscitation rates in this study are concordant with recently published rates for 22- to 25- week infants from the California Perinatal Quality Care Collaborative (CPQCC) [29].

This increase in active resuscitation has been observed nationally [39], particularly at the limit of viability where national resuscitation rates are higher than those in California. This is concordant with the ACOG guidance to consider antenatal corticosteroids at 22 weeks [40]. Recent data from the Neonatal Research Network (NRN), Vermont Oxford Network, and National Center for Health Statistics demonstrated active treatment rates of 30–36% at 22 weeks, 76–88% at 23 weeks, 98% at 24 weeks, and 99% at 25 weeks [6, 8, 18].

Survival after active resuscitation increased in this study with increasing gestational age with the most substantial change occurring over the periviable period (survival to hospital discharge was 33% at 22 weeks and increased to 82% at 25 weeks). As survival in the setting of extreme prematurity requires active resuscitation, survival data must be interpreted with the associated resuscitation practice variation in mind [17]. Survival rates in this study are comparable to a recent study from the NRN that used comparable methods to define active treatment and survival endpoints [6].

This study examined major morbidity in survivors to hospital discharge. In infants born at 22 weeks, 94% of those that survived to hospital discharge survived with an ICD code for at least one major morbidity. This decreased to 42% of infants by 28 weeks. Across the two study epochs, the percentage of infants born at 28 weeks surviving to hospital discharge without major morbidity increased significantly. Conversely, the same was not seen in infants 22–27 weeks. This is likely due to the limited 9-year timeframe of the study. Survival and survival without major morbidity increased across gestational ages 23–28 weeks in comparison to a previous California cohort study (2007–2011), although this study assessed major morbidity out to 1 year of age instead of hospital discharge [30]. Additionally, the national trend is towards decreased mortality and morbidity over a longer time frame [5, 6].

The most common major morbidities across all gestational ages in this cohort were sepsis and BPD. Higher rates of sepsis reported in this study in comparison to another studies [5, 6] are likely explained by reliance on ICD codes rather than cultures. Nevertheless, a significant decrease in the burden of sepsis was seen in the latter half of the study period for infants 25–28 weeks. This pattern has been previously reported in the literature [5, 41]. Conversely, a significant increase in 23- to 25-week infants affected by BPD was observed across the two epochs of this study. There is significant heterogeneity in BPD rates in the literature based on definition and site [42], but previous studies have also observed an increase in rates of BPD over time [5, 6] making it unique among major morbidities affecting extremely preterm infants.

The strengths of this study include the large sample size and population-based dataset inclusive of all live born infants ≤28 weeks across the state of California. Additionally, this study comprehensively reports rates of active resuscitation, survival and mortality for gestational ages 22–28 weeks with clear denominators, which is critical to using this data to guide counseling and practice. Limitations of the study include the exclusion of just over five thousand infants given the inability to link maternal and infant records and the reliance on ICD codes. ICD codes do not capture certain clinical details such stage of NEC, grade of BPD, or culture proven sepsis. Other clinical practices including administration of antenatal corticosteroids and delayed cord clamping were also unavailable due to the nature of the data. Additionally, although we cannot account for any effect from the change in the ICD classification system from the ninth to the tenth revision in 2015, we expect that it was minimal given the specificity of diagnostic and procedure codes chosen. Finally, neurodevelopmental outcomes are outside the scope of this study but critical to understand for a comprehensive perspective of long-term health outcomes in this population.

## Conclusion

This population-based study of preterm infants ≤28 weeks in California demonstrated increased active resuscitation and survival as well as decreased morbidity with increasing gestational age at birth. Over the study period, increased active resuscitation of periviable infants and increased morbidity free survival of 28-week infants was observed. Nevertheless, survival after extremely preterm birth was associated with significant morbidity with the prevalence of sepsis and BPD being notably high and both linked to poorer neurodevelopmental outcomes in early childhood [43, 44]. These trends may inform counseling around management of pregnancy and delivery at extremely preterm gestation with recognition of the limitations of population-based, gestational age predictions for individual infants and the important of family centered, shared decision making [45]. Further study of racial and ethnic as well as socioeconomic disparities in resuscitation practices, survival and morbidity is needed to improve outcomes in this vulnerable population.

### Supplementary information

Appendix

## Data Availability

The data that support the findings of this study are available from the California Department of Health Care Access and Information (https://hcai.ca.gov/data-and-reports/request-data/) and California Vital Statistics (https://www.cdph.ca.gov/Programs/CHSI/Pages/Data-Applications.aspx), but restrictions apply to the availability of these data, which were used under California Committee for the Protection of Human Subjects IRB for the current study, and so are not publicly available.
