# Supplementary material for: Resuscitation, survival and morbidity of extremely preterm infants in California 2011–2019
Source: J Perinatol. 2023 Sep 9;44(2):209–16. doi: 10.1038/s41372-023-01774-6 (PMC10844092; doi:10.1038/s41372-023-01774-6)
Supplement: Supplementary file 1 — Appendix [file 41372_2023_1774_MOESM1_ESM.docx]

Appendix 1- International Classification of Diseases Codes

|  | ICD-9 | ICD-10 | Vital statistics indication |
| --- | --- | --- | --- |
| Cesarean delivery | Maternal diagnostic code: 669.7  OR  Infant diagnostic code 763.4  OR maternal procedure code 74.0, 74.1, 74.2, 74.4, or 74.9 | Maternal diagnostic code: O82  OR  Infant diagnostic code P034  OR maternal procedure code 10D00Z0, 10D00Z1, or 10D00Z2 | X |
| Preexisting diabetes | Maternal diagnostic code: 648.0, 249, or 250 | Maternal diagnostic code: O24.0, O24.1, O24.2, O24.3, E10, E11, E12, E13, or E14 | X |
| Gestational diabetes | Maternal diagnostic code: 648.8 | Maternal diagnostic code: O24.4 | X |
| Preexisting hypertension | Maternal diagnostic code: 642.0, 642.1, or 642.2 | Maternal diagnostic code: O10 | X |
| Gestational hypertension | Maternal diagnostic code: 642.3 | Maternal diagnostic code: O13 | X |
| Preeclampsia | Maternal diagnostic code: 642.4, 642.5, 642.6, or 642.7 | Maternal diagnostic code: O11, O14.0, O14.1, O14.2, O14.9, or O15 | X |
| Chorioamnionitis | Maternal diagnostic code: 658.5  OR  Infant diagnostic code: 762.7 | Maternal diagnostic code: O41.1  OR  Infant diagnostic code: P02.7 | X |
| Resuscitation | Infant procedure code: 99.6, 93.9, 96.7, 94.04, or 96.05 | Infant procedure code: 5A09, 5A19, 0BH17EZ, or 5A2204Z |  |
| Intraventricular hemorrhage (IVH) grade III or IV | Infant diagnostic code: 772.13 or 772.14 | Infant diagnostic code: P52.2 |  |
| Periventricular leukomalacia (PVL) | Infant diagnostic code: 779.7 | Infant diagnostic code: P91.2 |  |
| Necrotizing enterocolitis (NEC) | Infant diagnostic code: 777.5 | Infant diagnostic code: P77 |  |
| Bronchopulmonary dysplasia (BPD) | Infant diagnostic code: 770.7 | Infant diagnostic code: P27 |  |
| Retinopathy of prematurity requiring intervention | Infant diagnostic code: 362.2 AND procedure 14.2, 14.5, or 14.7 | Infant diagnostic code: H35.1 AND procedure code with: 08 at first and second position AND 4,5, A,B, E, F, G or H at 4th position |  |
| Sepsis | Infant diagnostic code: 771.81 | Infant diagnostic code: P36 |  |
